# Roles of the anterior cingulate cortex and medial thalamus in short-term and long-term aversive information processing

**DOI:** 10.1186/1744-8069-6-42

**Published:** 2010-07-23

**Authors:** Sin-Chee Chai, Jen-Chuang Kung, Bai-Chuang Shyu

**Affiliations:** 1 Institute of Biomedical Sciences, Academia Sinica, Taipei 11529, Taiwan; 2 Department of Human Development, Tzu-Chi University, Hualien 97004, Taiwan

## Abstract

**Background:**

The anterior cingulate cortex (ACC) and medial thalamus (MT) are two of the main components of the medial pain pathway that subserve the affective aspect of pain. The hypothesis of the present study was that the ACC is involved in short-term aversive information processing and that the MT is critical for encoding unconditioned nociceptive information. The roles of these two components in short-term and long-term aversive information processing was investigated using a step-through inhibitory avoidance task.

**Results:**

Behavioral training began 1 week after surgery, in which radiofrequency lesions of the ACC or MT were performed. The retention tests were conducted 30 s (short-term) or 24 h (long-term) after training. Pretraining radiofrequency lesions of the ACC impaired performance in the 30 s, but not 24 h, retention test. Microinfusions of lidocaine into the ACC immediately after training impaired performance in the retention test conducted 10 min later. Pretraining radiofrequency lesions of the MT impaired performance in both the 30 s and 24 h retention tests. However, posttraining, but not pretest, microinfusions of lidocaine into the MT impaired performance in the 24 h retention test.

**Conclusions:**

These results suggest that the ACC may play an important role in short-term, but not long-term, nociceptive information processing. In contrast, the MT may be important for the consolidation of nociceptive information storage.

## Background

In rodents, area 24 of the anterior cingulate cortex (ACC) is a part of the medial prefrontal cortex, together with areas 25 and 32, and is implicated in the cognitive and emotional aspects of nociception [1-3]. Neuroimaging studies in humans have demonstrated ACC activation during noxious stimulation [4]. Behavioral studies in experimental animals have demonstrated that the ACC mediates responses to inflammatory pain [5,6], neuropathic pain [7], pain in the formalin test [8], and formalin-induced conditioned place avoidance [9,10]. These studies support the hypothesis that the ACC is involved in the processing of affective nociceptive information [11-14]. The ACC has been reported to be involved in the processing of both sensory nociceptive information and the anticipation of painful stimuli [1]. Therefore, a link between nociceptive stimuli (unconditioned stimulus, US) and anticipatory stimuli (conditioned stimulus, CS) may occur in this region [15]. Recent studies from our laboratory have explored the functional role of the ACC in nociceptive emotional learning. We found that lesions of the ACC reduce the association between a neutral cue and noxious thermal responses [16]. Furthermore, lesions of the medial prefrontal cortex block several types of short-term memory, working memory, and trace memory tasks [17-19].

The medial thalamus (MT) is part of the medial pain pathway that projects to the ACC and other prefrontal cortical areas and has been implicated in nociceptive information processing and memory function [20-24]. Electrical stimulation of the MT has been shown to evoke neural responses in the amygdala, an area implicated in emotional memory formation [25]. We have shown that short-term plasticity, such as paired-pulse facilitation, is evoked in the ACC by stimulation of the MT, suggesting that nociceptive information in the MT is transmitted to the ACC, and this may act to mediate short-term nociceptive information processing [26-29]. Lesions of the MT impaired delayed alternation in monkeys [30], trace eye blink conditioning in rabbits [31], active avoidance tasks in rats [32,33], and spatial memory tasks in rats [34,35].

Altogether, these studies suggest that the ACC and MT are involved in nociceptive information processing, but they might have additional functions involving aversive memory. The working hypotheses of the present study were that the ACC is involved in short-term aversive information processing, and the MT is critical for encoding unconditioned nociceptive information. We tested these hypotheses using the inhibitory avoidance task. First, ACC-lesioned rats were tested in a 30 s retention task to evaluate short-term information storage and in a 24 h retention task to evaluate long-term storage function. The ACC was also reversibly and transiently deactivated with lidocaine to study the temporal effects of inactivation. Second, we examined the role of the MT in the same nociceptive task. We found that lesions of the ACC impaired performance in the 30 s, but not 24 h, retention test, and infusions of lidocaine into the ACC immediately after training impaired performance in the retention test conducted 10 min later. Furthermore, pretraining lesions of the MT impaired performance in both the 30 s and 24 h retention tests. However, posttraining, but not pretest, infusions of lidocaine into the MT impaired performance in the 24 h retention test.

## Results

### Effects of ACC lesions

**Figure 1:**
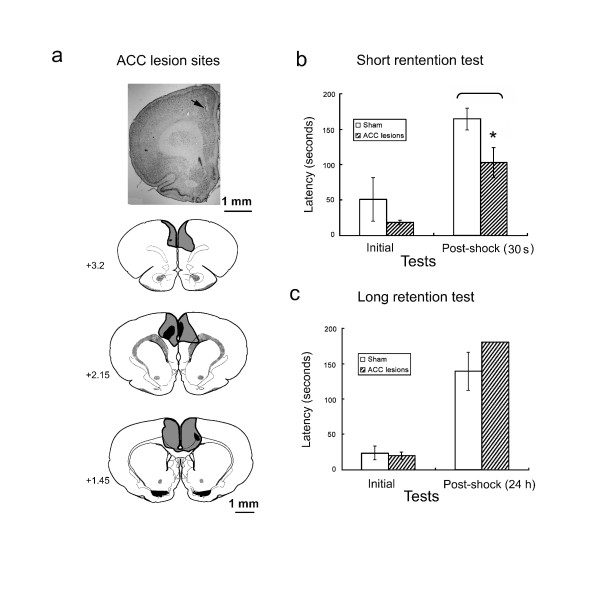
**Effects of ACC lesion on the initial and retention latencies tested in the inhibitory avoidance task**. **a**. A typical example of histology illustrating the lesion site (arrow). Drawing of radiofrequency lesions of the ACC sustained by rats in all experiments were composed in a series of coronal planes. Atlas templates of this and the following figures were adapted from Swanson [52]. The lighter areas indicate the maximum extent of all lesions, and darker areas indicate the minimum damage sustained by any rat. **b**. Mean latency in seconds (± SE) to enter the dark compartment in ACC sham and ACC lesion rats tested before and 30 s after footshock training. Fisher's LSD test showed a significant difference in latency between the ACC sham and ACC lesion rats tested 30 s after footshock (*p < 0.05) but no difference in initial latency. **c**. Mean latency in seconds (± SE) to enter the dark compartment in ACC sham and ACC lesion rats tested before and 24 h after electrical footshock training.

To investigate the role of the ACC in the inhibitory avoidance task, we first evaluate the retention latency between sham-and ACC-lesioned groups. The extent of the lesioned area in the ACC are shown in Fig. 1a. A large medial portion of the dorsal anterior cingulate was ablated. Some rats in this group also sustained damage to the medial precentral and prelimbic areas. No lesions extended to the infralimbic or dorsal peduncular areas.

Naive rats usually enter the dark compartment 10-20 s after being initially placed in the light compartment. The latency to enter the dark compartment was not significantly different for the ACC-lesioned group compared with the sham group. Retention latency was recorded for both the short-term and long-term retention period (Fig. 1b and 1c). An increase in retention latency indicates a delayed response after pairing the dark compartment with footshock, and this was observed in sham rats. In the short retention test (30 s interval), the increase in retention latency was suppressed in the ACC-lesioned group (Fig. 1b). In the long retention test (24 h interval), the retention latency was not suppressed in the ACC-lesioned group (Fig. 1c). A 2 × 2 analysis of variance (ANOVA) of the short retention test groups indicated significant effects of group (*F*_1,15 _= 4.82, *p *< 0.05) and test session (*F*_1,15 _= 27.62, *p *< 0.01) but no group × test session interaction. *Post hoc *comparisons using Fisher's Least Significant Difference (LSD) test revealed a significant difference in retention latency between the sham- and ACC-lesioned groups (*t *= 2.3, *p *< 0.05) tested 30 s later but no difference in initial latency. In the long retention test, the latency reached the cutoff time in the ACC-lesioned group. The ANOVA indicated no effect of group (*F*_1,17 _= 0.99, *p *> 0.05), a significant effect of test session (*F*_1,17 _= 109.16, *p *< 0.01), and no group × test session interaction. Therefore, ACC lesions appeared to diminish the delayed response, but only for the retention test conducted immediately, but not 24 h after, the footshock training.

### Effects of posttraining microinfusions of lidocaine into the ACC

**Figure 2:**
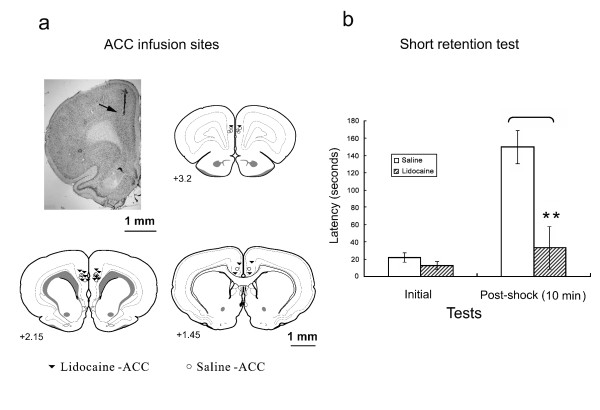
**Effects of microinfusion of lidocaine into the ACC on the initial and retention latencies tested in the inhibitory avoidance task**. **a**. Illustration of infusion cannula placements (arrow) in a histological coronal section. Placements shown are from all rats included in the final analysis (Saline, open circles; Lidocaine, filled inverted triangles). **b**. Lidocaine infusion into the ACC immediately after behavioral training disrupted retention of inhibitory avoidance learning tested 10 min later. Fisher's LSD test showed a significant difference between the saline and lidocaine microinfusion rats in the retention test 10 min after footshock (**p < 0.01).

Fig. 2a shows the cannula tip placements. Unsuccessful infusions resulted in the removal of four rats from the statistical analysis, yielding the following final group sizes: ACC-saline (*n *= 8), ACC-lidocaine (*n *= 8). Cannula placements were symmetrical throughout the rostrocaudal extent of the ACC and did not differ between groups.

Permanent radiofrequency lesions of the ACC did not affect performance in the long-term (24 h) retention test. Thus, only the short-term (10 min) retention test was conducted in the ACC experiments using lidocaine. Fig. 2b shows that the retention latency increased compared with the initial latency in the saline group, but not in the lidocaine infusion group. However, the increase was attenuated by lidocaine infusion immediately after footshock training. A 2 × 2 ANOVA showed significant effects of test session (*F*_1,16 _= 15.70, *p *< 0.01) and group (*F*_1,16 _= 22.56, *p *< 0.01) and a significant group × test session interaction (*F*_1,16 _= 11.00, *p *< 0.01). *Post hoc *comparisons using Fisher's LSD test revealed a significant difference between the sham and ACC-Lidocaine groups in the 10 min retention test (*t *= 5.11, *p *< 0.01) but not in the initial test (*t *= 0.42).

### Effects of MT lesions

**Figure 3:**
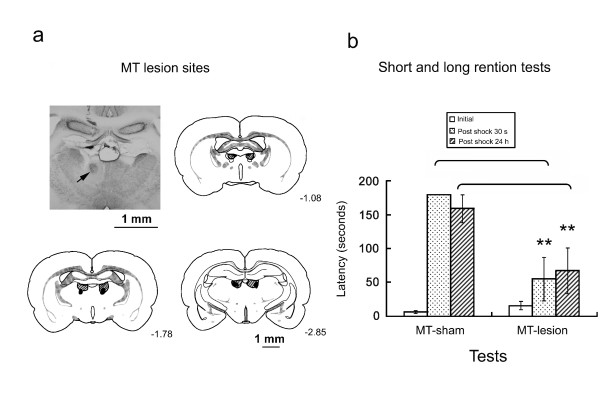
**Effects of MT lesion on the initial and retention latencies tested in the inhibitory avoidance task**. **a**. A typical example of histology illustrating the lesion site (arrow). Drawing of radiofrequency lesions of the MT sustained by rats in all experiments were composed in a series of coronal planes. The lighter areas indicate the maximum extent of all lesions, and the darker areas indicate the minimum damage sustained by any rat. **b**. Mean latency in seconds (± SE) to enter the dark compartment by MT Sham and MT lesion rats tested before, 30 s after (short retention test), and 24 h after (long retention test) footshock training.

To investigate the role of the MT in the inhibitory avoidance task, the same procedure was conducted in sham-and MT-lesioned groups. The lesions of the MT are shown in Fig. 3a. Only rats that had sustained bilateral MT lesions were retained for further statistical analysis. Two rats were excluded from this group because their lesions were unilateral and too small. For all of the subjects, the lesions were fairly circumscribed, including the entire MT and parts of the stria medullaris of the thalamus, habenula, reuniens lateral nucleus, and paraventricular nucleus.

Fig. 3b shows the latency to enter the dark compartment before and after receiving footshocks. In both the short-term and long-term retention test, the MT sham group entered the dark compartment after the shock with longer latencies than the MT-lesioned group. Significant differences were observed between the sham- and MT-lesioned groups (*F*_1,16 _= 9.52, *p *< 0.05), as well as for the three latency (*F*_2,16 _= 26.27, *p *< 0.01) and for their interactions (*F*_2,16 _= 8.93, *p *< 0.01). Fisher's LSD *post hoc t*-test showed significant differences between the sham- and MT-lesioned groups when they were tested 30 s (*t *= 3.93, *p *< 0.01) and 24 h (*t *= 5.34, *p *< 0.01) later. These findings suggest that the MT critically mediates the processing and storage of nociceptive information whenever these nociceptive memories are retrieved.

### Effects of microinfusions of lidocaine into the MT

**Figure 4:**
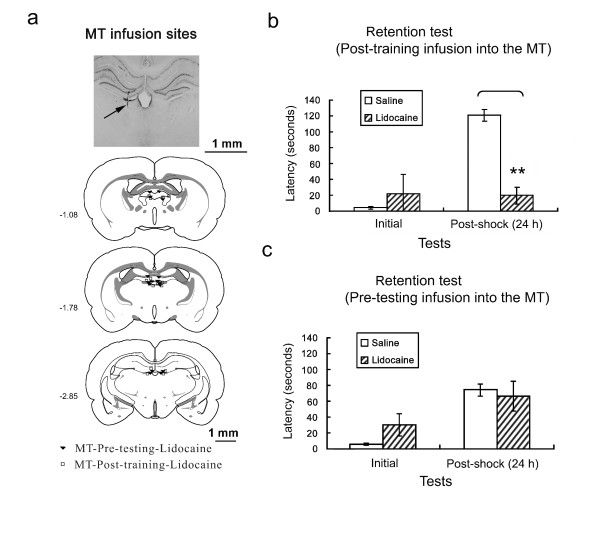
**Effects of microinfusion of lidocaine into the MT on the initial and retention latencies tested in the inhibitory avoidance task**. **a**. Illustration of infusion cannula placements (arrow) in a histological section of the medial thalamus. Placements shown are from all rats included in the final analysis (MT-pretest-lidocaine, filled inverted triangles; MT-posttraining-lidocaine, open squares). **b**. Lidocaine infusion into the MT immediate after behavioral training disrupted retention of inhibitory avoidance learning conducted 24 h later. Fisher's LSD test showed a significant difference between posttraining infusions of saline and lidocaine in the MT group in the 24 h retention test (**p < 0.01). **c**. Lidocaine infusion into the MT immediately before the 24 h retention test had no effect on the retention of inhibitory avoidance learning tested 24 h later.

Fig. 4a shows the infusion cannula tip placements for all rats included in the analysis. An unsuccessful infusion resulted in the removal of one rat from the analysis, yielding final group sizes of *n *= 8 for both the MT-posttraining-lidocaine and MT-pretest-lidocaine groups. Cannula placements were symmetrical throughout the rostrocaudal extent of the MT and did not consistently differ between groups.

Fig. 4b shows that the effects of lidocaine infusions into the MT immediately after footshock training. The ANOVA revealed significant main effects of footshock (*F*_1,11 _= 21.12, *p *< 0.01) and lidocaine infusion (*F*_1,11 _= 11.50, *p *= 0.01) and a significant footshock × lidocaine infusion interaction (*F*_1,11 _= 22.90, *p *< 0.01). Fisher's LSD *post hoc t*-test showed significant differences between the posttraining saline and lidocaine infusion groups (*t *= 7.63, *p *< 0.01). This test also showed a significant difference between posttraining infusions of saline and lidocaine into the MT in the 24 h retention test. Fig. 4c shows the effects of saline and lidocaine infusions into the MT before the 24 h retention test. The ANOVA revealed a significant main effect of footshock (*F*_1,14 _= 24.28, *p *< 0.01), but no effect of lidocaine infusion and no footshock × lidocaine infusion interaction.

## Discussion

The results indicate that lesions of the ACC and MT differentially affect performance in the inhibitory avoidance task. The fact that the lesions and temporary deactivation of the ACC only impair performance in the short-term retention test but not long-term retention test supports the hypothesis that the ACC is involved in short-term aversive information processing. The results from the MT lesions show that they impaired performance in both the 30 s and 24 h retention tests. The results from the intra-MT lidocaine infusions administered immediately after behavioral training showed impairment in the 24 h retention test. This implies that the MT is essential for encoding unconditioned nociceptive information.

### Effects of ACC lesions

The present ACC lesion results have two implications. First, the ACC is involved in the mediation of short-term inhibitory avoidance information processing, and this labile information lasts only for a short period of time after training. Second, the information processing and memory of the inhibitory avoidance task may involve more than one memory system [36,37]. Lesions of the ACC impaired performance in the short-term, but not long-term, inhibitory avoidance tasks, suggesting that nociceptive information involving electrical footshocks was processed by a system that includes the ACC and other brain areas. The ACC may be activated immediately after footshock to mediate conditioning, whereas non-ACC systems may be involved in the mediation of a similar type of nociceptive information processing that was reactivated after longer durations and required longer consolidation or storage periods. Therefore, the same type of memory could still be remembered after a longer period. This finding is consistent with the possibility that memories with different properties or characteristics are mediated by different areas in the brain [38]. Lesions of the ACC did not block long-term inhibitory avoidance learning, suggesting that the ACC may not be involved in the retrieval of aversive learning information.

Posttraining microinfusions of lidocaine into the ACC impaired performance in the 10 min retention task, supporting the hypothesis that the ACC may be involved in short-term information processing in this task. Although we did not test the rats 24 h later, the analysis of a group of rats that receive pretraining ACC lesions and are tested 24 h later may provide a partial answer to the acquisition, consolidation, and retention questions. The ACC may not be involved in information processing at any of the stages for long-term inhibitory avoidance tasks. This finding is consistent with a series of conditioned freezing experiments using either context or tone as the CS. Sacchetti et al. [39] showed that posttraining tetrodotoxin inactivation of the prefrontal, frontal, and parietal cortices did not block freezing tested 72 h after CS-US conditioning. However, in an earlier study, Sacchetti et al. [40] showed that pretraining lidocaine inactivation of the prefrontal, frontal, and parietal cortices did not block the freezing tested 3 h after CS-US conditioning. All pretraining microinfusions impaired the conditioned freezing tested 72 h after conditioning, and microinfusions of lidocaine failed to block the acquisition or expression of short-term memory, but it did block long-term memory. Although these results suggest that short-term and long-term nociceptive information storage may be mediated by different parallel memory systems in the brain, these findings appear to be opposite to our results and Saccheti et al. [39]. However, their interpretation was that the frontal cortex is involved only in the earliest, initial consolidation of the fear response to the CS. Similar to the prefrontal cortex and frontal cortex, the ACC appears to be more critical to the acquisition of fear responses than to subsequent memory processing. The main difference between our studies and the previous study is that different behaviors were measured. An operant behavior (i.e., entering a dark compartment) was measured in our experiments, and Pavlovian conditioning (i.e., freezing in response to a CS) was measured in the previous study. Brain lesions that increase freezing in response to the CS could have increased the latency to enter the dark compartment, but lesions that block freezing might not necessarily reduce the latency to enter the dark compartment.

### ACC and nociceptive information processing

Vaccarino and Melzack [41] showed that injections of lidocaine into the anterior cingulum bundle significantly reduced formalin-induced pain but not shock-induced pain, suggesting that this area is involved in the perception of tonic pain but not phasic pain. Recent evidence showed that lesions of the ACC blocked the conditioned place aversion induced by hindpaw formalin injections, suggesting that the ACC is necessary for the acquisition or expression of conditioned avoidance information processing elicited by a nociceptive stimulus [9,10]. The present findings appear to be inconsistent with these previous studies showing that long-term aversion is impaired by ACC lesions. The main difference between these previous experiments and the present study is that the US used in the present study was electrical footshock, and the US used in the previous experiments was formalin. A recent study showed that formalin, but not shock, induced conditioned place aversion, which was blocked by ACC lesions [42]. This finding further suggests that the ACC may play differential roles in shock-and formalin-induced aversive learning. However, the role played by the ACC in pain information processing is still not clearly defined and may not be critically involved in the processing of nociceptive information in the present study. If ACC lesions principally impaired sensory pain information, then a deficit in inhibitory avoidance should be observed at all time-points; however, this was not the case. In contrast, the ACC is critical for information processing underlying the recognition of pain-predictive cues. In a recent study from our laboratory, rats were trained to associate a tone with laser stimulation that produced acute pain. Rats exhibited increased movements at the onset of the tone. These conditioned responses were eliminated by ACC lesions when tested immediately after the laser pain conditioning training [16]. Neuronal responses to a pain-related CS have been reported, indicating that the ACC is involved not only in the sensory aspect of pain, but also in pain conditioning [15]. Similar results were obtained in a study using rabbits. Conditioned stimulus-elicited changes in multiple-unit activity were recorded in the medial prefrontal cortex, and these changes progressively increased over tone-shock association sessions [2]. Furthermore, this neural activity was attenuated by pretraining limbic thalamic lesions [35]. A recent study showed that infusions of muscimol or AP5 into the ACC immediately, 90 min, or 180 min after inhibitory avoidance training attenuated retention, but this did not occur when they were given 24 h after training [43]. This finding is consistent with the present results, indicating that ACC lesions do not appear to affect inhibitory avoidance when the avoidance test is conducted 24 h later.

### Effects of MT lesions

Lesions of the MT blocked inhibitory avoidance, both 30 s and 24 h after footshock training, and suggest that the MT is required for inhibitory avoidance information processing. The MT is specifically related to the affective component of pain, and its possible function may be to convey unconditioned nociceptive signals to higher cortical areas. When the footshock is delivered after the animal enters the dark compartment, in addition to spinal-based reflexive movements, one stream of footshock information may be processed from peripheral receptors via the MT to the somatosensory cortex and limbic systems. If the MT is damaged, then no further processing of nociceptive information would occur. Therefore, an association between the CS and nociception will not be formed. The peripheral pain responses of foot withdrawal observed during the delivery of an electric footshock may be a spinal reflex that does not involve the central nervous system.

Reversible lesions of the MT immediately after behavioral training, but not before behavioral testing, disrupted the retention of the 24 h inhibitory avoidance. This finding suggests that the MT is crucial for conveying nociceptive information to cortical or limbic areas. Another possible explanation is that inactivation of the MT interrupts the consolidation of nociceptive memory. The nociceptive information may be transmitted to the thalamus, and the outputs from this information are then projected to the ACC and other limbic areas. The MT-ACC system involves fast, short-term plasticity related to emotional learning [26]. The other slow pathway may involve systems including the MT-lateral nuclei of the amygdala and hippocampal systems.

The present study did not include pretraining lidocaine infusions because lidocaine-induced inactivation of the MT can last for approximately 30 min. Pretest lidocaine infusions had no effects on the retention of inhibitory avoidance, and the effect of pretraining infusions might be the same as the permanent radiofrequency lesions.

Considerable evidence suggests that the MT is involved in one or more aspects of information processing and memory. Hunt and Aggleton [34] found that lesions of the mediodorsal thalamic nuclei impaired acquisition in a nonspatial object recognition task. However, the same lesions failed to disrupt working memory in a radial maze task, but the lesions did affect other processes that interact with information processing tasks, reflected in the level of general activity. Alexinsky [44] further showed that ibotenic acid lesions of the mediodorsal thalamus induced a mild and reversible deficit in complex radial maze tasks, but not working memory or the priming effect. Smith et al. [35] showed in rabbits that lesions of the MT blocked information processing related to neural responses to task-relevant stimuli for the cingulate cortex. These findings suggest that cingulothalamic circuitry is important for avoidance information processing.

### Prefrontal cortex and working memory

A substantial amount of literature shows that the prefrontal cortex plays a prominent role in working memory [45-47]. Recent experiments have demonstrated that ACC subregions are involved in working memory for egocentric responses but not working memory for spatial locations or food reward value [48,49]. The involvement of the prefrontal cortex in working memory is characterized by activation of some part of the area and occurs when sensory information in the absence of a stimulus must be retained for proper execution of a response over a short period of time. Although there is no consensus about the duration of working memory, a 15 s delay between behavioral training and retrieval trials has been used in several other studies. Ragozzino and Kesner [50] showed that rats could be trained before surgery on a delayed match-to-sample task that involved the memory of a 90° right or left turn. Lesions of the ACC significantly decreased scores in the 10 s delay condition compared with presurgery levels [50]. In contrast, Kolb et al. [51] showed that medial prefrontal cortex lesions impaired working memory of visual objects or patterns observed at delays of 15 s or longer, but not at shorter delays. This evidence suggests that the effects of prefrontal cortex lesions on working memory are related to the actual duration of the delays used in the experiment.

## Conclusions

The present findings indicate that the ACC may be important for nociceptive short-term information, but not long-term storage. Moreover, the MT may be an essential gateway allowing the acquisition and consolidation of both types of nociceptive information storage. These findings provide strong support for the involvement of medial pain pathways in complex information processing and emotional processes. Furthermore, the results provide some support for the existence of functional specialization of the ACC and MT.

## Methods

### Subjects

Male Sprague-Dawley rats were purchased from the National Animal Breeding Center, Taipei, Taiwan, and weighed 250-300 g. Rats were housed three per cage on a 12 h/12 h light/dark cycle (lights on at 0800 h). Food and water were available *ad libitum*. All behavioral testing was performed during the light phase of the cycle. The rats were handled at least 3 days before performing the behavioral training and were allowed to adapt to the experimental room. Forty-nine rats were assigned to the ACC experiments, and 55 rats were assigned to the MT experiments. All experiments were performed in accordance with the guidelines established by the Academia Sinica Institutional Animal Care and Utilization Committee.

### Surgical Procedure

#### Radiofrequency lesions

The rat was anesthetized (450 mg/kg chloral hydrate, i.p.) and placed with the skull flat in a sterile stereotaxic apparatus (David Kopf Instruments, Tujunga, CA, USA). For lesions of the ACC, the skull was exposed, and four holes were drilled 2.5 mm and 1.5 mm anterior to bregma and 0.5 mm lateral to the midline bilaterally. For lesions of the MT, two holes were drilled 2.5 mm posterior to bregma and 0.5 mm lateral to the midline bilaterally. A lesion was made at the electrode tip with a Lesion Generator System (Model RFG-4A, Radionics Inc., Burlington, MA, USA). The electrode was repeatedly penetrated through the four holes and advanced to 1.5 mm ventral to dura, 1.5 mm anterior to bregma, and 2.5 mm ventral to dura, 2.5 mm anterior to bregma for the ACC lesion. For the MT lesion, the electrode was penetrated through the two holes and advanced to 5 mm ventral to dura. After each electrode penetration, the temperature at the electrode tip was maintained at 80°C for 30 s. The rats received bilateral radiofrequency lesions of the ACC (*n *= 19) and MT (*n *= 12). The incision was then closed, and penicillin (500,000 units, 0.2 ml, i.p.) was administered. Rats were kept warm until recovery from anesthesia and were then returned to their home cages. The subjects in the sham condition (ACC, *n *= 10; MT, *n *= 5) underwent identical procedures, with the exception that no heat was delivered through the electrode tip. The subjects were weighed daily, and recovery was monitored for 1 week before behavioral testing.

#### Cannulation and microinfusion

The rat was treated with atropine sulfate, anesthetized with chloral hydrate (450 mg/kg, i.p.), and mounted in the stereotaxic apparatus. The head was positioned to place bregma and lambda in the same horizontal plane. The skull was exposed, and small holes were drilled through the skull for bilateral placement of stainless steel guide cannulae (23 gauge, 10 mm length) into the ACC (2.0 mm anterior to bregma, 0.5 mm lateral to bregma, 1.5 mm ventral to dura) or MT (2.5 mm posterior to bregma, 0.5 mm lateral to bregma bilaterally, 5 mm ventral to dura). The cannula was affixed by three screws attached to the skull with dental cement. Stainless steel obturators (30 gauge, 10 mm length; Small Parts Inc., Miami Lakes, FL, USA) were placed into the guide cannula. After surgical operation, penicillin was administered intraperitoneally, and the behavioral procedure began 1 week later. The infusions were made with a 30 gauge needle connected by polyethylene tubing to a 1 ml Terumo syringe. The needle tip protruded 1 mm beyond the end of the guide cannula. A total of 1 μl for each site was infused over 60 s, and the needle was left in place for an additional 60 s before being withdrawn. The subjects were weighed daily, and recovery was monitored for 1 week before behavioral testing.

### Behavioral apparatus

The apparatus consisted of two compartments, one light and one dark, separated by a sliding guillotine door with a 10 cm × 9.5 cm opening. The light compartment was a 31 cm × 22 cm × 21 cm box with wood chips on the floor and a clear Plexiglas top. The black compartment was an opaque 28 cm × 18 cm × 21 cm box with black plastic wall coverings. The black compartment had a grid floor with parallel copper rods spaced 1.5 cm apart. A shock generator that provided square wave electrical current (Model 2100, A-M Systems Inc., Carlsborg, Washington, USA) was connected to the rods.

### Procedures

The light/dark step-through inhibitory avoidance task consisted of the following. After 5 min of handling, the rat was placed in the light compartment facing away from the guillotine door. Once the rat entered with all four legs into the dark compartment, the duration was recorded as the initial latency, and the door was immediately closed. After a 3 s delay, an inescapable footshock (0.5 mA for 3 s) was delivered, and the rat was returned to its home cage. For the ACC lesion experiment, the ACC-lesion/short-term group (*n *= 12) was tested 30 s later, and the ACC-lesion/long-term group (*n *= 7) was tested 24 h later. The same procedure was applied to the MT lesion (*n *= 12) and MT Sham (*n *= 5) groups. The rat was reintroduced to the light compartment, and the latency to enter the dark compartment was recorded as the retention latency. The cutoff time was 180 s.

All rats with microinfusions of lidocaine into the brain were tested 10 min, rather than 30 s, after the electrical footshock training in the short-term memory group. This was because of technical restrictions while infusing lidocaine into the brain. The entire microinfusion procedure lasted approximately 5 min; therefore, testing the rat 30 s after the behavioral training was impossible.

**Figure 5:**
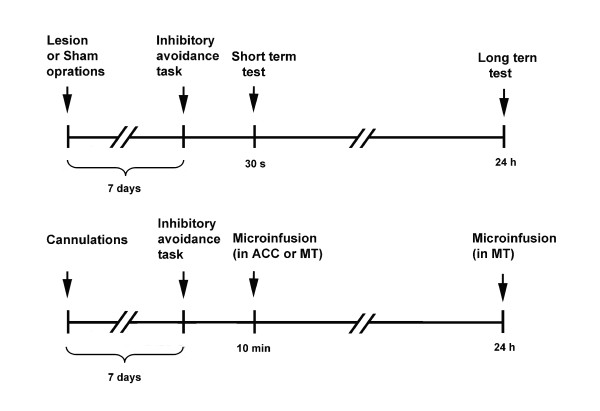
**Schematic diagram of the experimental procedures**. The procedures for surgical operations and the short-and long-term tests in the ACC and MT lesioned groups are illustrated in the upper panel. The procedures for cannulations and microinfusions to evaluate posttraining and pretest effects in the ACC and MT groups are illustrated in the lower panel.

For the lidocaine infusion experiment, rats were randomly assigned to the following groups: ACC-lidocaine (*n *= 10), ACC-saline (*n *= 10), MT posttraining saline (*n *= 5), MT posttraining lidocaine (*n *= 12), MT pretest saline (*n *= 9), and MT pretest lidocaine (*n *= 12). Lidocaine (2 mg/kg, 1 μl) or an equivalent volume of saline was infused immediately after the rats received the footshock and were then removed from the inhibitory avoidance chamber. The experimental procedures are illustrated in Fig. 5.

### Histology

Shortly after the behavioral tests, the rats were deeply anesthetized with chloral hydrate (600 mg/kg, i.p.) and perfused with isotonic saline followed by a 10% formalin solution. The brain was removed and placed in a 10% formalin/sucrose solution until the brain sank. It was then frozen and sectioned along the coronal plane (100 μm). The size and location of each lesion were compared with the appropriate brain sections in Swanson's rat brain atlas [52].

## Competing interests

The authors declare that they have no competing interests.

## Authors' contributions

SCC participated in the design of the study, conducted the experiments, analyzed the data, and drafted the manuscript. JCK participated in the discussion of the experimental results and suggested the experiments. BCS conceived the study, participated in its design and coordination, and wrote the manuscript. All authors read and approved the final manuscript.
